# Association of Immune-Related Adverse Events, Hospitalization, and Therapy Resumption With Survival Among Patients With Metastatic Melanoma Receiving Single-Agent or Combination Immunotherapy

**DOI:** 10.1001/jamanetworkopen.2022.45596

**Published:** 2022-12-08

**Authors:** Alexander S. Watson, Siddhartha Goutam, Igor Stukalin, Benjamin W. Ewanchuk, Michael Sander, Daniel E. Meyers, Aliyah Pabani, Winson Y. Cheung, Daniel Y. C. Heng, Tina Cheng, Jose G. Monzon, Vishal Navani

**Affiliations:** 1 Tom Baker Cancer Centre, Department of Oncology, University of Calgary, Calgary, Alberta, Canada; 2 Faculty of Medicine and Dentistry, University of Alberta, Edmonton, Alberta, Canada; 3 Department of Medicine, University of Calgary, Calgary, Alberta, Canada; 4 Cumming School of Medicine, University of Calgary, Calgary, Alberta, Canada

## Abstract

**Importance:**

Immune-related adverse events (irAEs) due to immune checkpoint blockade (ICB) have been shown to be positively associated with survival. Among patients with metastatic melanoma, evidence supporting this association has been conflicting, while ipilimumab-nivolumab combination ICB has been examined only in small clinical cohorts.

**Objective:**

To examine the association between irAEs and survival among patients with metastatic melanoma, in particular for those receiving combination ICB.

**Design, Setting, and Participants:**

A retrospective cohort of 492 consecutive patients with metastatic melanoma treated with ICB at 2 tertiary and 4 regional cancer centers in Alberta, Canada, from August 1, 2013, to May 31, 2020, was observed. Patients were aged 18 years or older with metastatic melanoma agnostic to primary site, who received 1 or more doses of an anti–programmed cell death protein 1 agent as single or combination ICB. Clinically significant irAEs requiring systemic corticosteroids and/or treatment delay were captured. To minimize immortal time bias, only patients surviving 12 weeks after ICB initiation were included in survival analyses. Statistical analysis was conducted on December 10, 2021.

**Exposures:**

Development of irAEs requiring systemic corticosteroids and/or treatment delay.

**Main Outcomes and Measures:**

The primary outcome was overall survival (OS), with the association of irAE development with OS assessed via Kaplan-Meier and Cox proportional hazards regression analyses. The association of hospitalization for irAEs and ICB resumption after irAE with OS was examined.

**Results:**

Among 492 patients, the median age of those with irAEs was 61.8 years (IQR, 52.9-72.1 years), and the median age of those without irAEs was 65.5 years (IQR, 56.5-76.9 years), while sex distribution was comparable (137 of 198 men [69.2%] with irAEs vs 183 of 294 men [62.2%] without irAEs). There was an association between irAEs and OS both in the overall cohort (with irAEs: median OS, 56.3 months [95% CI, 38.2 months to not evaluable] vs without irAEs: median OS, 18.5 months [95% CI, 14.4-23.2 months]; *P* < .001) and in the 124 patients (25.2%) receiving combination ICB (with irAEs: median OS, 56.2 months [95% CI, 52.2 months to not evaluable] vs without irAEs: median OS, 19.0 months [95% CI, 6.6 months to not evaluable]; *P* < .001). Hospitalization for irAE did not alter this positive association with OS compared with outpatient treatment (median OS, not evaluable [95% CI, 31.5 months to not evaluable] vs median OS, 52.2 months [95% CI, 35.2 months to not evaluable]; *P* = .53), while resumption of ICB was associated with longer OS than not resuming ICB (median, 56.3 months [95% CI, 40.8 months to not evaluable] vs 31.5 months [95% CI, 21.0 months to not evaluable]; *P* = .009). A favorable independent association of irAEs with OS was confirmed in multivariable analysis (hazard ratio for death, 0.382 [95% CI, 0.254-0.576]; *P* < .001).

**Conclusions and Relevance:**

This study suggests an association between irAEs and OS for patients with metastatic melanoma, including those treated with combination ICB and those with severe irAEs requiring hospitalization. The potential benefit associated with ICB resumption after irAEs warrants further investigation.

## Key Points

**Question:**

Are immune-related adverse events associated with longer overall survival among patients with metastatic melanoma treated with combination immune checkpoint blockade?

**Findings:**

In this cohort study of 492 patients, those who experienced clinically significant immune-related adverse events had significantly longer median overall survival (56.3 months vs 18.5 months). This trend was maintained with combination immune checkpoint blockade and unaltered by hospitalization, while resumption of immunotherapy after the adverse event was associated with longer survival.

**Meaning:**

This study suggests that despite increased hospitalization, immune-related adverse events are associated with longer survival among patients receiving combination immune checkpoint blockade, while resuming immunotherapy may benefit selected patients.

## Teaser

This cohort study examines the association between immune-related adverse events and survival among patients with metastatic melanoma, in particular for those receiving combination immune checkpoint blockade.

## Introduction

Outcomes in metastatic melanoma have dramatically shifted since the introduction of antibodies targeting immune system checkpoints (immune checkpoint blockade [ICB]).^1^ In particular, dual blockade of both programmed cell death-1 and cytotoxic T-lymphocyte–associated antigen 4 (CTLA-4), with nivolumab and ipilimumab, respectively (combination ICB), generates durable responses and median overall survival (OS) beyond 5 years.^2,3^

Immune-related adverse events (irAEs) are common ICB toxic effects, with severe (grade 3 or higher) irAEs experienced by 59% of trial patients receiving combination ICB.^2,4^ These diverse autoimmune-mediated pathologies are thought to represent “bystander” attack on healthy tissues and require specialized management,^5^ with hospitalization for severe symptoms.^6^

Given their frequency, the association of irAEs with patient outcomes has been of particular interest. Retrospective data and meta-analyses for single-agent ICB across multiple tumor sites have largely observed improved objective response rates, progression-free survival, and OS among patients who develop irAEs.^7,8,9,10,11^ In metastatic melanoma, the association of irAEs with outcomes has been more variable.^7,12,13^ One proposed explanation may be immortal time bias, in particular in early studies.^13,14^ A second reason may be the weaker association of irAEs with OS among patients receiving anti–CTLA-4 therapy,^8,15^ creating uncertainty around combination ICB.^8^ Two recent analyses of combination ICB have supported an association between irAEs and survival^16,17^; however, patient numbers were small, and one of the studies was focused on early irAEs.^17^

Another factor to consider for combination ICB is the association of more severe irAEs with survival, given that hospitalization and longer duration of immunosuppression are often required.^5^ Again, discrepancy exists in the literature, with some studies suggesting a weaker association^9^ or even reduced OS for higher-grade irAEs among patients receiving single-agent ICB.^10^ In particular, after severe irAEs, the risk of recurrence with the reintroduction of ICB is significant; thus, understanding how ICB resumption is associated with outcomes is crucial.^17^ Prior analyses have shown nonsignificant trends toward longer OS but may have been underpowed^18^; clarifying these associations is important for discussions about patient care and clinical decision-making.

Here, we examine a multicenter cohort of patients with metastatic melanoma treated with single-agent and combination ICB. We characterize irAEs requiring systemic corticosteroids and/or delaying treatment and assess the association of irAEs, hospitalization for irAEs, and ICB resumption with survival outcomes.

## Methods

### Study Design

The Alberta Immunotherapy Database (AID) is a multicenter, province-wide observational cohort study. AID captures baseline demographic, histologic, clinical, laboratory, and imaging data using a standardized template.^19^ This study was reviewed and approved by the Health Research Ethics Board of Alberta–Cancer Committee, which waived patient consent given the low-risk, deidentified, retrospective nature of this work. Inclusion criteria were age 18 years or older, confirmed metastatic melanoma (agnostic to site of origin), and receipt of at least 1 cycle of ICB (single-agent nivolumab, pembrolizumab, or combined ipilimumab-nivolumab) at any line of therapy. Results are reported in accordance with the Strengthening the Reporting of Observational Studies in Epidemiology (STROBE) reporting guideline.

Objective imaging response was defined by investigator assessment, using RECIST (Response Evaluation Criteria in Solid Tumours), version 1.1 criteria.^20,21^ Prior to undertaking data analysis, treatment response was categorized into best overall response, classified as complete response, partial response, stable disease, and progressive disease. Patients received treatment between August 1, 2013, and May 31, 2020. Patients missing treatment duration data were excluded from the survival analysis.

### Outcomes of Interest

Our study evaluated patients with metastatic melanoma fitting the inclusion criteria, identifying irAEs and comparing patients with or without irAEs who received ICB. The primary end point of interest was OS, while the secondary outcome was time to next treatment (TTNT). We examined the association with those survival outcomes of baseline characteristics (demographic, disease, and treatment), irAE development, hospitalization for irAEs, and ICB resumption after an irAEs (defined as receiving ≥1 cycle of ICB after withholding ICB for irAEs, independent of progression).

**Figure 1.**
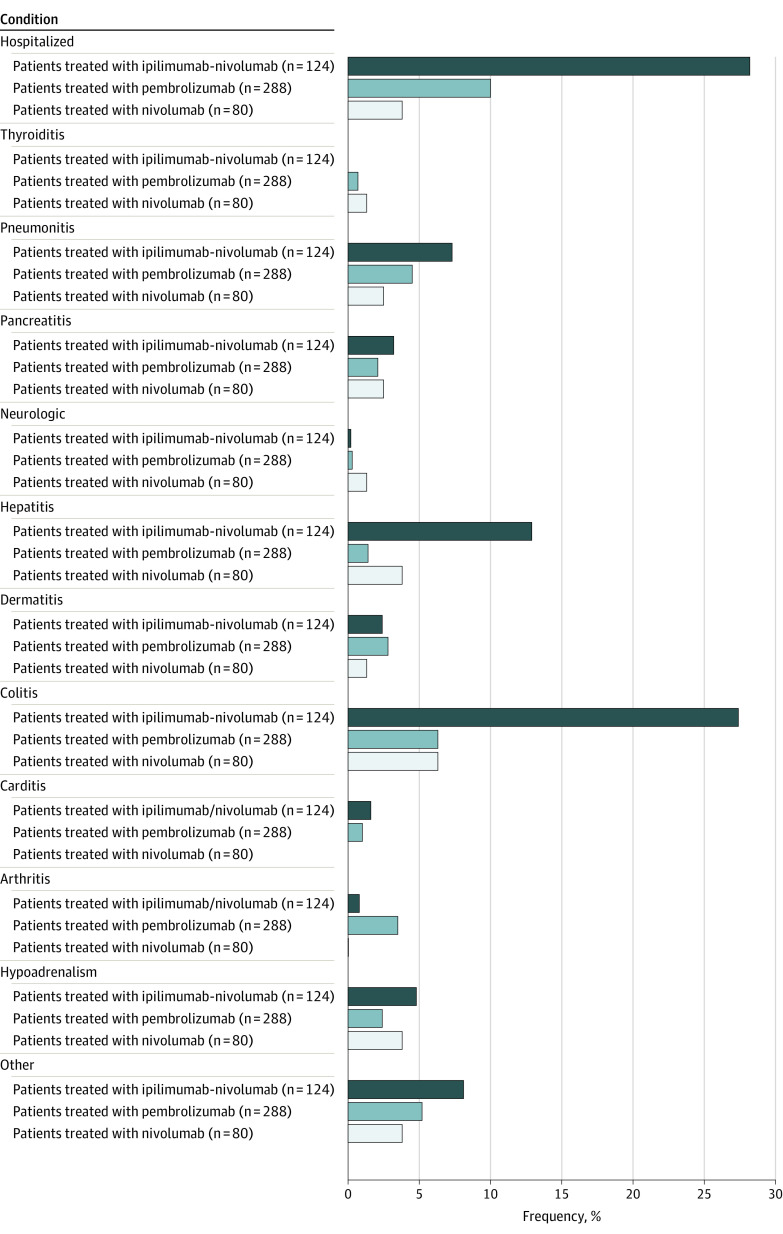
Relative Frequency and Type of Clinically Significant Immune-Related Adverse Events (irAEs) by Agent Received The irAEs included as “Other” (≤2 patients) include febrile syndrome, cytokine release syndrome, nephritis, myositis, polymyalgia, Sjögren syndrome, hypophysitis, vomiting syndrome, hemolytic anemia, bleeding, immune-related pancytopenia, uveitis or iritis, chorioretinitis, bullous pemphigoid, mucositis, and nonnecrotizing granulomatous inflammation.

Immune-related adverse events were identified based on clinician notes in the medical record at the time of development of the irAEs, with only clinically significant irAEs experienced by patients included, defined as grade 2 or higher per the Common Terminology Criteria for Adverse Events (CTCAE), version 5.0,^22^ requiring systemic corticosteroids and/or delay of ICB. If a patient developed multiple irAEs, the most clinically impactful irAE was used. Immune-related adverse events were grouped as per Figure 1, with irAEs identified on medical record review not fitting predefined categories recorded as “Other.”

### Statistical Analysis

Statistical analysis was conducted on December 10, 2021. Associations between irAE development and baseline characteristics were assessed using the χ^2^ test and the Fisher exact test. To minimize immortal time bias from patients with a poor prognosis, a 12-week landmark (corresponding to median time of irAE development) was implemented for OS and TTNT analyses; patients who died before 12 weeks from ICB initiation were excluded from Kaplan-Meier analyses (compared using the log-rank test). Overall survival was defined as time from commencement of ICB to date of death or censored last follow-up. Time to next treatment was defined as time from commencement of ICB to commencement of subsequent systemic therapy, death, or censored last follow-up. A multivariable Cox proportional hazards regression model of OS and TTNT was adjusted for significant (2-sided *P* ≤ .05) underlying differences in baseline characteristics, including ICB cycles received and combination vs single-agent ICB.

The case deletion method was used when missing data were encountered. All statistical tests were 2-sided, and results were deemed statistically significant at *P* ≤ .05, performed using SAS Cloud of Academics (SAS, version 9.4; SAS Institute Inc).

## Results

### Characteristics of Population, Disease, and Treatments Received

In total, 492 patients with metastatic melanoma received ICB during the study period, with baseline characteristics as outlined in Table 1; the median age for patients with irAEs was 61.8 years (IQR, 52.9-72.1 years) compared with 65.5 years (IQR, 56.5-76.9 years) for those without irAEs, with comparable sex distribution (137 of 198 men [69.2%] with irAEs vs 183 of 294 men without irAEs [62.2%]). By χ^2^ analysis, those with irAEs were more likely to be younger than 50 years (43 of 198 [21.7%] vs 41 of 294 [13.9%]; *P* = .02), have an Eastern Cooperative Oncology Group (ECOG) performance status of 0 (106 of 198 [53.5%] vs 93 of 293 [31.7%]; *P* < .001), and a normal albumin level (171 of 198 [86.4%] vs 220 of 294 [74.8%]; *P* = .002). Disease characteristics, such as histologic characteristics and *BRAF* (GenBank NG_007873) variant status, did not differ between those with and those without irAEs.

**Table 1.** Baseline Characteristics of Patients With Metastatic Melanoma With or Without Immune-Related Adverse Events^a^

Characteristics	Patients, No. (%) (N = 492)		*P* value
	Without irAEs (n = 294)	With irAEs (n = 198)	
Age, y			
≥50	253 (86.1)	155 (78.3)	.02^b^
<50	41 (13.9)	43 (21.7)	
Median (IQR)	65.5 (56.5-76.9)	61.8 (52.9-72.1)	NA
BMI, median (IQR)	28.1 (24.3-31.6)	28.5 (24.3-31.5)	NA
Sex			
Male	183 (62.2)	137 (69.2)	.11
Female	111 (37.8)	61 (30.8)	
ECOG performance status at ICB start (n = 491)			
0	93/293 (31.7)	106 (53.5)	<.001^b^
1	153/293 (52.2)	72 (36.4)	
2	39/293 (13.3)	18 (9.1)	
3	8/293 (2.7)	2 (1.0)	
Hemoglobin			
Normal	190 (64.6)	143 (72.2)	.07
Below lower limit of normal	104 (35.4)	55 (27.8)	
Lactate dehydrogenase			
Normal	199 (67.7)	147 (74.2)	.12
Above upper limit of normal	95 (32.3)	51 (25.8)	
Albumin			
Normal	220 (74.8)	171 (86.4)	.002^b^
Below lower limit of normal	74 (25.2)	27 (13.6)	
Primary melanoma histologic characteristic			
Cutaneous	194 (66.0)	137 (69.2)	.50
Desmoplastic	6 (2.0)	2 (1.0)	
Mucosal	11 (3.7)	8 (4.0)	
Ocular	27 (9.2)	10 (5.1)	
Anal	7 (2.4)	2 (1.0)	
Vulval	6 (2.0)	2 (1.0)	
Ungual	2 (0.7)	1 (0.5)	
Muscle primary	1 (0.3)	0	
Unknown	40 (13.6)	36 (18.2)	
*BRAF* variant (n = 410)^c^			
Present	86/242 (35.5)	61/168 (36.3)	.87
Absent	156/242 (64.5)	107/168 (63.7)	
M stage			
M1a/b	141 (48.0)	98 (49.5)	.74
M1c/d	153 (52.0)	100 (50.5)	
Metastatic site (n = 246)^d^			
Brain	56/155 (19.1)	32/91 (16.2)	.41
Liver	99/155 (33.7)	59/91 (29.8)	.37
Treatment line first ICB (n = 490)			
First	190 (64.6)	159/196 (81.1)	.002^b^
Second	51 (17.4)	21/196 (10.7)	
Third	43 (14.6)	14/196 (2.9)	
Fourth or later	10 (3.4)	2/196 (1.0)	
ICB agent			
Nivolumab	56 (19.0)	24 (12.1)	<.001^b^
Pembrolizumab	201 (68.4)	87 (43.9)	
Ipilimumab-nivolumab	37 (12.6)	87 (43.9)	
Cycles received, median (range)	6 (3-13)	6 (3-14)	
Best observed response (n = 383)^e^			
Complete response	27/214 (12.6)	32/169 (18.9)	<.001^b^
Partial response	57/214 (26.6)	83/169 (49.1)	
Stable disease	35/214 (16.4)	23/169 (13.6)	
Progressive dsease	95/214 (44.4)	31/169 (18.3)	

Abbreviations: BMI, body mass index (calculated as weight in kilograms divided by height in meters squared); ECOG, Eastern Cooperative Oncology Group; ICB, immune checkpoint blockade; irAEs, immune-related adverse events; NA, not applicable.

^a^ Immune-related adverse events were considered only if they required systemic corticosteroids and/or treatment delay.

^b^ Significantly different between those who developed irAEs and those with no irAEs (at *P* ≤ .05).

^c^ *BRAF* variants were V600E (94.6% [122 of 129]) or V600K (5.4% [7 of 129]).

^d^ Patients in either group may have multiple metastatic sites.

^e^ Best observed response was categorized per Response Evaluation Criteria in Solid Tumours criteria.

A total of 288 patients received pembrolizumab as their first ICB agent, 80 received nivolumab, and 124 received combination ICB with ipilimumab-nivolumab. Those who developed irAEs were significantly more likely to have received first-line ICB (159 of 196 [81.1%] vs 190 of 294 [64.6%]; *P* = .002) and combination ICB (87 of 198 [43.9%] vs 37 of 294 [12.6%]; *P* < .001) and to have a complete or partial response (115 of 169 [68.0%] vs 84 of 214 [39.3%]; *P* < .001) (Table 1). A total of 93 patients (50% of patients with irAE meeting the survival landmark) who developed irAEs had ICB resumed later. The median follow-up was 36.6 months (95% CI, 33.6-38.3 months).

### Characterization of irAEs

Figure 1 demonstrates the relative frequency of clinically significant irAEs (grade 2 or more, requiring systemic corticosteroids and/or delay of treatment) experienced by patients in our cohort. The 2 single ICB agents, nivolumab and pembrolizumab, were comparable; the frequency of irAEs was 28.8% (23 of 80) and 30.2% (87 of 288), respectively, with colitis most common (nivolumab, 6.3% [5 of 80]; pembrolizumab, 6.3% [18 of 288]). A total of 70.2% of patients (87 of 124) treated with combination ICB experienced irAEs, including a 27.4% rate of colitis (34 of 124), a 12.9% rate of hepatitis (16 of 124), and a 7.3% rate of pneumonitis (9 of 124).

Hospitalization rates for irAE were significantly higher in the combination ICB group than in the nivolumab and pembrolizumab groups (28.2% [35 of 124] vs 3.8% [3 of 80] vs 10.1% [29 of 288]; *P* < .001 for combination vs single-agent ICB). Almost all patients with carditis were hospitalized (4 of 5 [80.0%]), followed by those with pneumonitis (14 of 24 [58.3%]) and those with neurologic irAEs (2 of 4 [50.0%]) (eTable in the Supplement). Median times to irAE development are shown in eTable in the Supplement; carditis was noted at the earliest time point after therapy initiation (0.88 months [IQR, 0.85-0.89 months]), while neurologic irAEs and pancreatitis were highly variable and could develop with substantial delay (neurologic irAEs, 7.4 months [IQR, 3.3-18.8 months]; pancreatitis, 7.5 months [IQR, 1.2-15.2 months]).

### Survival Outcomes After irAE Development, Hospitalization, and ICB Resumption

**Figure 2.**
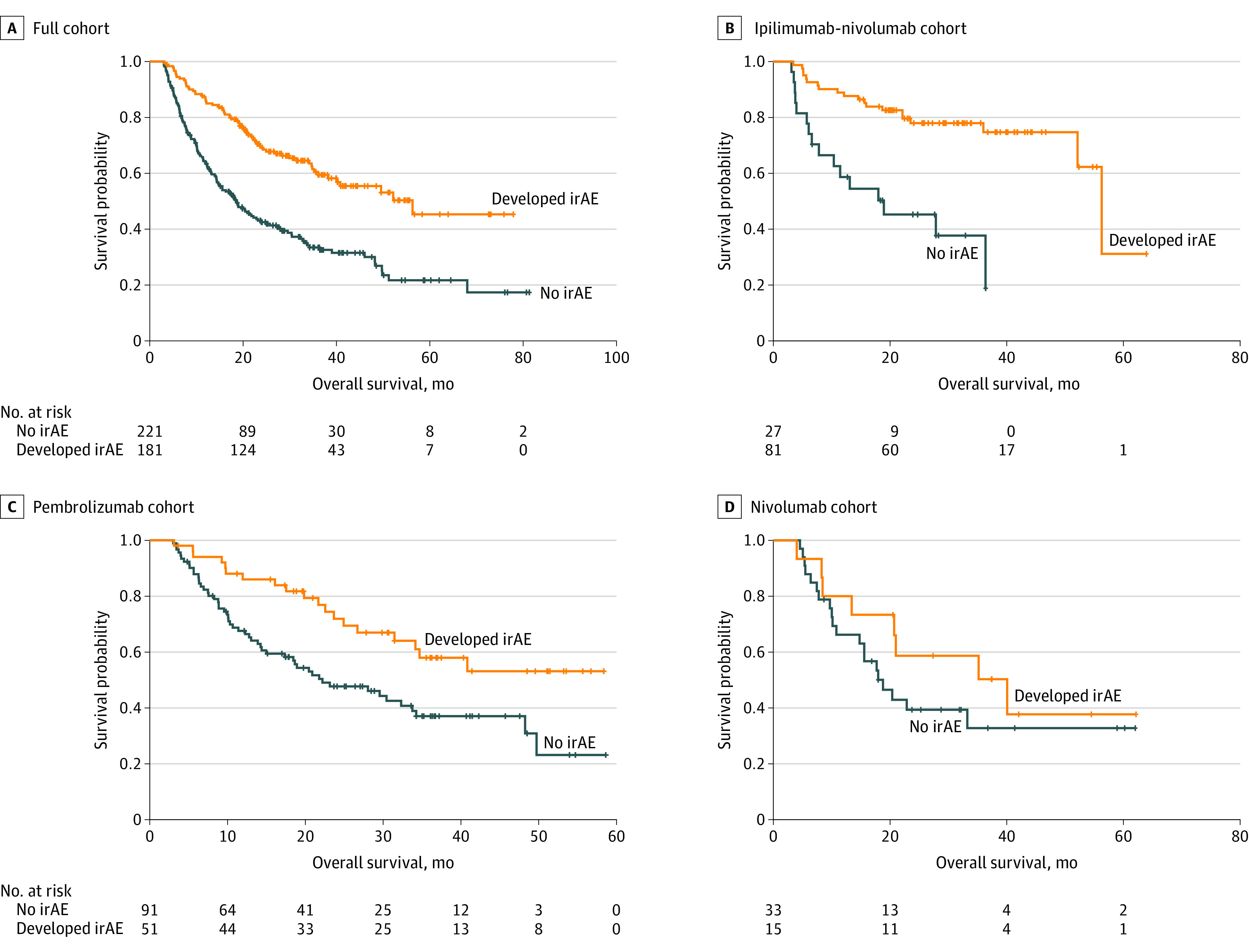
Median Overall Survival (OS) for Patients With Metastatic Melanoma Surviving to at Least 12 Weeks A, Full cohort of patients with or without immune-related adverse events (irAEs) (median OS, 56.3 months [95% CI, 38.2 months to not evaluable] vs 18.5 months [95% CI, 14.4-23.2 months]; *P* < .001). B, Patients treated with ipilimumab-nivolumab combination immune checkpoint blockade therapy (median OS, 56.2 months [95% CI, 52.2 months to not evaluable] vs 19.0 months [95% CI, 6.6 months to not evaluable]; *P* < .001). C, Patients treated with pembrolizumab (median OS, not evaluable [95% CI, 31.5 months to not evaluable] vs 22.2 months [95% CI, 14.4-33.8 months]; *P* = .004). D, Patients treated with nivolumab (median OS, 40.1 months [95% CI, 8.4 months to not evaluable] vs 18.8 months [95% CI, 10.8 months to not evaluable]; *P* = .30).

In 12-week landmark analysis, patients who developed an irAE while receiving ICB had significantly longer median OS compared with those who did not (median, 56.3 months [95% CI, 38.2 months to not evaluable] vs 18.5 months [95% CI, 14.4-23.2 months]; *P* < .001) (Figure 2). Examining individual agents (Figure 2B-D), significance was maintained for those treated with combination ICB (median OS, 56.2 months [95% CI, 52.2 months to not evaluable] vs 19.0 months [95% CI, 6.6 months to not evaluable]; *P* < .001) and pembrolizumab (median OS, not evaluable [95% CI, 31.5 months to not evaluable] vs 22.2 months [95% CI, 14.4-33.8 months]; *P* = .004), but not for those treated with nivolumab (median OS, 40.1 months [95% CI, 8.4 months to not evaluable] vs 18.8 months [95% CI, 10.8 months to not evaluable]; *P* = .30).

**Figure 3.**
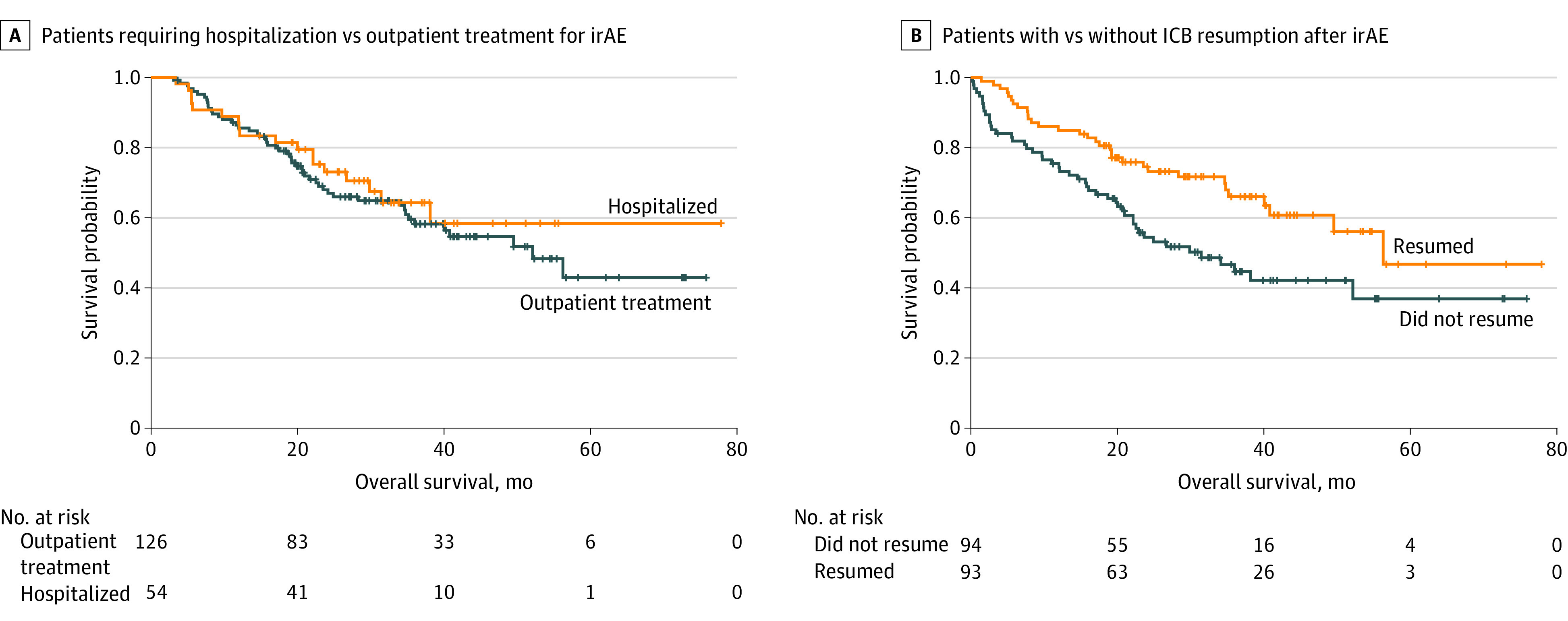
Median Overall Survival (OS) for Patients With Metastatic Melanoma Surviving to at Least 12 Weeks A, Patients requiring hospitalization vs outpatient-only treatment for immune-related adverse events (irAEs) (median OS, not evaluable [95% CI, 31.5 months to not evaluable] vs 52.2 months [95% CI, 35.2 months to not evaluable]; *P* = .53). B, Patients with or without immune checkpoint blockade (ICB) resumption after irAE recovery (median OS, 56.3 months [95% CI, 40.8 months to not evaluable] vs 31.5 months [95% CI, 21.0 months to not evaluable]; *P* = .009).

Among patients who developed irAEs, those who required hospitalization had comparable median OS with those treated as outpatients (not evaluable [95% CI, 31.5 months to not evaluable] vs 52.2 months [95% CI, 35.2 months to not evaluable]; *P* = .53) (Figure 3A). Resumption of ICB after irAEs was associated with longer median OS compared with not resuming ICB (56.3 months [95% CI, 40.8 months to not evaluable] vs 31.5 months [95% CI, 21.0 months to not evaluable]; *P* = .009) (Figure 3B). There was no difference observed in OS between those with early (<12 weeks) irAEs and those with late (≥12 weeks) irAEs, nor was differential OS observed among those developing the 3 most common irAEs (colitis, hepatitis, and pneumonitis).

Similar results were seen in TTNT analysis, with the development of irAEs associated with longer TTNT (median TTNT, 49.6 months [95% CI, 21.1 months to not evaluable] vs 12.9 months [95% CI, 10.0-15.7 months]; *P* < .001) (eFigure, A, in the Supplement), which did not change if the irAEs required hospitalization vs outpatient treatment (median TTNT, not evaluable [95% CI, 20.0 months to not evaluable] vs 34.2 months [95% CI, 19.8 months to not evaluable]; *P* = .20) (eFigure, B, in the Supplement).

### Variables Associated With OS by Cox Proportional Hazards Regression Analysis

To further assess the association between potential prognostic factors and OS among those surviving to 12 weeks, Cox proportional hazards regression analysis was performed. On univariable analysis, along with established prognostic variables, such as ECOG performance status, lactate dehydrogenase level, and presence of stage M1c/d disease, clinically significant irAE development was associated with OS (hazard ratio [HR] for death, 0.427 [95% CI, 0.321-0.569]; *P* < .001), as was hospitalization due to irAEs (HR for death, 0.498 [95% CI, 0.307-0.808]; *P* = .005), combination ICB treatment (HR for death, 0.560 [95% CI, 0.400-0.790]; *P* < .001), first-line treatment with ICB (HR for death with second-line treatment vs first-line treatment, 1.812 [95% CI, 1.260-2.608]; *P* = .001), and additional ICB cycles (HR for death, 0.958 [95% CI, 0.944-0.971]; *P* < .001) (Table 2). On adjusted multivariable analysis of all significant univariable factors, including adjustment for ICB cycles received and treatment with combination ICB, irAE development remained independently associated with longer OS (HR for death, 0.382 [95% CI, 0.254-0.576]; *P* < .001).

**Table 2.** Univariable and Multivariable Cox Proportional Hazards Regression Analysis of Factors Associated With Overall Survival

Factor	Univariable analysis		Multivariable analysis	
	Death, HR (95% CI)	*P* value	Death, HR (95% CI)	*P* value
irAE	0.427 (0.321-0.569)	<.001^a^	0.382 (0.254-0.576)	<.001^a^
Hospitalized with irAE	0.498 (0.307-0.808)	.005^a^	0.596 (0.328-1.081)	.09
Early irAE (<12 wk)	1.373 (0.854-2.208)	.19	NA	NA
Combination ICB	0.560 (0.400-0.790)	<.001^a^	0.688 (0.434-1.091)	.11
Treatment line ICB				
Second line (vs first)	1.812 (1.260-2.608)	.001^a^	1.608 (1.002-2.580)	.05^a^
Third line (vs first)	1.663 (1.125-2.458)	.01^a^	1.118 (0.698-1.789)	.64
Fourth line (vs first)	1.779 (0.868-3.646)	.12	2.181 (0.978-4.866)	.06
ICB cycle	0.958 (0.944-0.971)	<.001^a^	0.940 (0.923-0.957)	<.001^a^
Aged <50 y	0.789 (0.544-1.143)	.21	NA	NA
Male sex	1.045 (0.791-1.382)	.76	NA	NA
ECOG performance status at ICB start				
1 (vs 0)	1.687 (1.267-2.246)	<.001^a^	0.971 (0.679-1.389)	.87
2 (vs 0)	1.796 (1.050-3.072)	.03^a^	0.772 (0.359-1.658)	.51
3 (vs 0)	1.525 (0.558-4.169)	.41	0.432 (0.127-1.473)	.18
LDH above upper limit of normal	2.159 (1.619-2.880)	<.001^a^	2.074 (1.463-2.939)	<.001^a^
Albumin below lower limit of normal	1.837 (1.297-2.602)	<.001^a^	1.791 (1.102-2.910)	.02^a^
Hemoglobin below lower limit of normal	1.613 (1.213-2.145)	.001^a^	1.048 (0.707-1.554)	.82
Stage M1c/d	1.785 (1.362-2.340)	<.001^a^	1.604 (1.159-2.222)	.004^a^
Cutaneous histologic characteristic	0.855 (0.461-1.588)	.62	NA	NA
*BRAF* variant^b^	1.086 (0.798-1.478)	.60	NA	NA

Abbreviations: ECOG, Eastern Cooperative Oncology Group; HR, hazard ratio; ICB, immune checkpoint blockade; irAE, immune-related adverse event; LDH, lactate dehydrogenase.

^a^ Significant at 2-tailed *P* ≤ .05.

^b^ *BRAF* variants were V600E (94.6% [122 of 129]) or V600K (5.4% [7 of 129]).

## Discussion

In this multicenter, retrospective population-based cohort study, longer OS among patients with metastatic melanoma experiencing irAEs was observed for those receiving single-agent and combination ICB, despite the latter’s higher rates of hospitalization for irAEs. Our results suggest that hospitalization for more severe irAEs does not alter the positive association with survival, while resumption of ICB after irAEs may be associated with improved outcomes. This is, to our knowledge, the largest examination of the incidence of irAEs and their association with survival outcomes for patients receiving combination ICB in a clinical setting in the literature to date.

The proposed association between irAEs and outcomes is consistent with the mechanism of ICB; an immune response that is sensitive to ICB antitumor priming is more prone to bystander effects in healthy tissues.^7^ Characteristics more common among patients who developed irAEs in our study, namely younger age, normal albumin level or nutritional status, and robust ECOG performance status, are consistent with prior data^23^ and can be associated with a less-senescent immune system.^24^ The median ICB cycle number was similar between those with and those without irAEs, as were disease variables, supporting the notion that irAEs related to a given ICB are most likely associated with host factors. Our inability to find an irAE-OS association for nivolumab is likely owing to the small sample size of that cohort (n = 79).

The median time to development of irAEs in our cohort and the frequency of irAEs with each ICB regimen were consistent with published values.^1,2,11,14,17,25^ Our method of capturing clinically significant irAEs (systemic corticosteroids and/or treatment delay) would exclude grade 1 toxic effects, topically treated dermatitis, and mild hypothyroidism; hence, our values are best compared with grade 2 or higher irAEs. Hospitalization rates were likewise similar to published clinical experiences.^6^

Despite being a clinical indicator of ICB activity, irAEs requiring corticosteroids, treatment delays, and/or hospitalization have the potential to blunt therapeutic outcomes. Given that prior data around grade 3 or 4 toxic effects, more common with combination ICB, have suggested weaker or no associations with OS,^9,10,26^ and that anti-CTLA-4 therapies have more frequent toxic effects with weaker outcome associations,^8,9^ there were reasons for uncertainty around the use of combination ICB. In the largest clinical cohort to date, our data support a positive association with OS for patients who develop clinically significant irAEs while receiving combination ICB, in keeping with other reported series.^16,17^

Among patients with irAEs overall, hospitalization for irAEs (guideline management for grade 3-4 toxic effects) did not alter the positive survival association, which was maintained in univariable regression, although not meeting multivariable significance. These findings build on prior data from our group^27^ and other larger meta-analyses^17,28^ to help inform clinician-patient discussions after the development of severe irAEs. The equipoise in the literature around the association of irAE severity with outcomes^10^ may be due to differing hospitalization thresholds and management patterns globally.

Another factor may be decisions around ICB resumption, with our analysis supporting a potential association between resumption of ICB after clinically significant irAEs and OS. This observation, in keeping with trends observed in a recent analysis of a smaller population,^18^ suggests that, among carefully selected patients who have recovered from a significant irAE, further ICB therapy is not only safe but may improve outcomes. However, retrospective assessment of such clinical decisions in particular are vulnerable to selection bias from uncaptured confounders. Ksienski et al^17^ found that discontinuation of combination ICB due to irAEs was not associated with survival; however, most of those patients still received maintenance nivolumab. Further investigation, ideally prospective, of ICB resumption is required.

### Strengths and Limitations

This study has some strengths. One advantage to our study is the use of a 12-week landmark to minimize distortion associations from patients with poor prognosis dying prior to the median time of irAE development, proposed as a confounder in early irAE studies.^13,14^ Grade 5 irAEs were not specifically captured in our retrospective database owing to limitations of the medical records; however, their rarity (no irAE deaths at <12 weeks in the Checkmate 067 trial^2^) supports most excluded patients dying from melanoma. Our multivariable Cox proportional hazards regression analysis was adjusted for the cycles of ICB received, as well as for combination ICB treatment, increasing confidence that the irAE-OS association is independent of the agent used.

This study also has some limitations, including those associated with its retrospective nature, including unaccounted biases in patient selection and irAE management. Retrospective gathering of subjective assessments, such as ECOG performance status, are reliant on those assessments being included in the medical record through prior notes and are best interpreted as a spectrum rather than discrete values. Our irAE definition also varied from some prior studies, and the focus on severe or clinically impactful irAEs may have influenced our findings. The association of irAEs with quality of life is an important consideration for care not captured by survival analyses.

## Conclusions

The data in our cohort study support a favorable survival outcome among patients with metastatic melanoma who develop clinically impactful irAEs while receiving ICB, including combination ICB. Clinicians may be reassured that hospitalization for more severe irAEs is not negatively associated with OS, supporting care discussions and decisions with patients. Resumption of ICB may be associated with benefit for certain patients after an irAE; however, given confounders, prospective study will be needed.

## Notes

Supplement. **eTable.** Hospitalization Rates and Time to Development for Immune-Related Adverse Events **eFigure.** Time-To-Next-Treatment (TTNT) for Metastatic Melanoma Patients Treated With Immune Checkpoint Blockage (ICB) Surviving to at Least 12 Weeks
