# Supplementary material for: Association of Immune-Related Adverse Events, Hospitalization, and Therapy Resumption With Survival Among Patients With Metastatic Melanoma Receiving Single-Agent or Combination Immunotherapy
Source: JAMA Netw Open. 2022 Dec 8;5(12):e2245596. doi: 10.1001/jamanetworkopen.2022.45596 (PMC9856439; doi:10.1001/jamanetworkopen.2022.45596)
Supplement: Supplement. — eTable. Hospitalization Rates and Time to Development for Immune-Related Adverse Events eFigure. Time-To-Next-Treatment (TTNT) for Metastatic Melanoma Patients Treated With Immune Checkpoint Blockage (ICB) Surviving to at Least 12 Weeks [file jamanetwopen-e2245596-s001.pdf]

Supplementary Online Content

Watson AS, Goutam S, Stukalin I, et al. Association of immune-related adverse events, hospitalization, and therapy resumption with survival among patients with metastatic melanoma receiving single-agent or combination immunotherapy. *JAMA Netw Open.* 2022;5(12):e2245596. doi:10.1001/jamanetworkopen.2022.45596

eTable. Hospitalization Rates and Time to Development for Immune-Related Adverse Events
eFigure. Time-To-Next-Treatment (TTNT) for Metastatic Melanoma Patients Treated With Immune Checkpoint Blockage (ICB) Surviving to at Least 12 Weeks

This supplementary material has been provided by the authors to give readers additional information about their work.

© 2022 Watson AS et al. *JAMA Network Open.*

eTable. Hospitalization Rates and Time to Development for Immune-Related Adverse Events[a]

Type of irAE[a]	N[c]	Hospitalized (% of irAE)	Median Time (months) to irAE development (IQR)
Adrenal	16	4 (25%)	3.92 (2.18)
Arthritis	13	1 (8%)	5.54 (2.83)
Carditis	5	4 (80%)	0.88 (0.03)
Colitis	57	25 (44%)	1.97 (1.97)
Dermatitis[b]	12	0	1.38 (5.13)
Hepatitis	23	3 (13%)	1.77 (1.68)
Neuro	4	2 (50%)	7.36 (15.47)
Other	28	11 (39%)	2.99 (3.56)
Pancreatitis	12	3 (25%)	7.52 (13.96)
Pneumonitis	24	14 (58%)	2.56 (3.60)
Thyroiditis	3	0	3.32 (2.89)
Overall	195	67 (34%)	2.56 (3.94)

Abbreviations: Immune-related Adverse Event (irAE), Interquartile Range (IQR)

a. irAE requiring systemic steroids and/or treatment delay
b. Dermatitis requiring oral steroids
c. 3 cases deleted for missing time data

© 2022 Watson AS et al. *JAMA Network Open.*

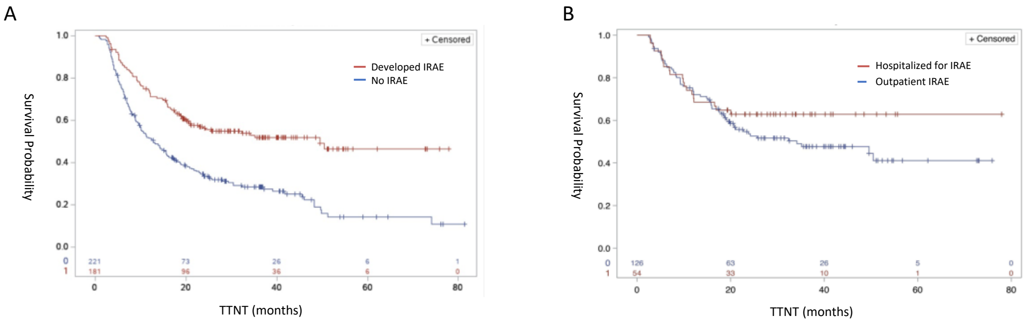

eFigure. Time-To-Next-Treatment (TTNT) for Metastatic Melanoma Patients Treated With Immune Checkpoint Blockage (ICB) Surviving to at Least 12 Weeks

A) With or Without immune-related Adverse Events (irAE) (median [95% CI] 49.6 [21.1-Not evaluable(NE)] vs 12.9 [10.0-15.7] months, p<0.001) and B) Requiring Hospitalization vs Outpatient only treatment for irAE (NE [20.0-NE] vs 34.2 [19.8-NE] months, p=0.20)

© 2022 Watson AS et al. *JAMA Network Open.*
